# Manufacture and Quality Control of Human Umbilical Cord-Derived Mesenchymal Stem Cell Sheets for Clinical Use

**DOI:** 10.3390/cells11172732

**Published:** 2022-09-01

**Authors:** Juan Wang, Shuang Gao, Yufei Zhao, Taibing Fan, Mingkui Zhang, Dehua Chang

**Affiliations:** 1 BOE Regenerative Medicine Technology Co., Ltd., Beijing 100015, China; 2 Children Heart Center, Fuwai Central China Cardiovascular Hospital, Zhengzhou 450018, China; 3 Heart Center, First Hospital of Tsinghua University, Beijing 100016, China; 4 Department of Cell Therapy in Regenerative Medicine, The University of Tokyo Hospital, Tokyo 113-8655, Japan

**Keywords:** umbilical cord mesenchymal stem cell sheet, large-scale production, preservation

## Abstract

Human umbilical cord-derived mesenchymal stem cell (UC−MSC) sheets have attracted much attention in cell therapy. However, the culture media and coating matrix used for the preparation of UC−MSC sheets have not been safe enough to comply with current clinical drug standards. Moreover, the UC−MSC sheet preservation systems developed before did not comply with Good Manufacturing Practice (GMP) regulations. In this study, the culture medium and coating matrix were developed for UC−MSC sheet production to comply with clinical drug standards. Additionally, the GMP-compliant preservation solution and method for the UC−MSC sheet were developed. Then, quality standards of the UC−MSC sheet were formulated according to national and international regulations for drugs. Finally, the production process of UC−MSC sheets on a large scale was standardized, and three batches of trial production were conducted and tested to meet the established quality standards. This research provides the possibility for clinical trials of UC−MSC sheet products in the development stage of new drugs and lays the foundation for industrial large-scale production after the new drug is launched.

## 1. Introduction

Human umbilical cord-derived mesenchymal stem cells (UC−MSCs) are considered a promising cell source for autologous and allogeneic cell therapy because of their relatively low immunogenicity compared to mesenchymal stem cells (MSCs) from adult counterparts, in which HLA-ABC is expressed very weakly [1,2]. UC−MSCs have strong biological activity and differentiation capacity even after repeated amplification [3]. UC−MSCs are also compliant with standard amplification in potentially large quantities due to their fast self-renewing capacity [4,5], and UC−MSC banks can be established to cater to market demand after drug approval. Therefore, UC−MSCs are considered a promising cell source for clinical cell therapy [6,7].

In cell therapy, MSC transplantation is usually carried out by local or transcatheter injection of cell suspension. However, generally, only 10–20% of injected cells are available at the injured area within a few hours or days after delivery, and only a few cells actively engraft in the affected tissue [8]. In addition, attached cells are digested by enzymes to produce a cell suspension, which destroys cellular connexins and greatly reduces cell activity and function [9]. Therefore, cell suspension injection causes significant loss and death of cells and uneven local distribution, which greatly reduces the expected therapeutic effects [8]. Cell sheet technology, developed by Prof. Okano’s team [9,10], eliminates the problem of retention, helps to retain cells, and provides the appropriate lifespan for the transplanted MSCs. Using this technology, cell sheets were prepared and they improved the cardiac function in mouse [10] and porcine [11] myocardial infarction models.

For extensive clinical promotion, robust cell sheet production, preservation, and quality control systems conforming to Good Manufacturing Practice (GMP) regulations are essential elements of successful cell therapy.

To date, the reported cell sheet-forming medium has been either fetal bovine serum (FBS)-containing media or serum-free mesenchymal stem cell culture media [12,13]. As a kind of exogenous macromolecule protein, bovine serum albumin (BSA) in FBS-containing media can cause allergic reactions. Exogenous growth factors or research-grade human/animal extracts—additives for supporting cell growth—in serum-free mesenchymal stem cell culture media could also cause safety risks in humans. For instance, excessive exogenous growth factor residues in cell therapy final products may pose a carcinogenic risk in humans. In addition, research-grade reagent residues in cell therapy final products do not meet the GMP regulations because no safety evaluations have been conducted in humans. Therefore, the serum-containing and serum-free media commonly used are not suitable for clinically safe cell sheet preparation.

Following production, the cell sheet products need to be transported to the clinical site that will administer the cells; therefore, the cell sheet preservation method and solution need to be developed. Several teams have reported a cell sheet preservation method and solution, but cultured cell sheets were used for preservation in all studies [14,15,16,17]. When reaching the clinical site, a series of complex operations, such as cell sheet detaching, were needed before usage, which would introduce high risks to the final product. Thus, the preservation methods were not in compliance with the GMP guidelines. 

In order to ensure consistency and quality controllability of the UC−MSC sheet products, a two-tiered cell bank system should be established and quality controlled as intermediate products in the production process according to the “ICH Q5D: Derivation and Characterization of Cell Substrates Used for Production of Biotechnological/Biological Products”. Generally, a two-tiered cell bank system includes master cell banks (MCBs) and working cell banks (WCBs), in which a batch of MCB is used to generate several batches of WCBs. Therefore, the final UC−MSC sheet products can be produced from the quality controlled WCBs to ensure batch-to-batch consistency.

In this study, the preparation and preservation technology for UC−MSC sheets, which complied with the GMP guidelines, was developed for use in clinical trials and preclinical studies. First, a safe UC−MSC sheet production technology was successfully developed to ensure drug safety and efficacy. Second, an effective UC−MSC sheet preservation technology was developed to ensure storage and transportation, from which UC−MSC sheets could be preserved for 24 h with cell viability greater than 70%. Third, quality standards involving product characteristics, safety, and functionality were established according to the “ICH Q5A: Viral Safety Evaluation of Biotechnology Products Derived from Cell Lines of Human or Animal Origin”, “ICH Q6B: Test Procedures and Acceptance Criteria for Biotechnological/Biological Products”, “Guiding Principles for Quality Control of Stem Cell Preparations and Preclinical Research”, “Guidelines for Cell Therapy Products and Evaluation Technology”, and our research results. Fourth, the production process of UC−MSC sheets on a large scale was standardized, including donor screening, cell bank establishment and quality control, and cell sheet production and quality control. Finally, three batches of trial production were conducted according to the standardized production process. All three batches of MCBs and WCBs in trial production met the established quality standards. UC−MSC sheets with tight cell connections and an intact extracellular matrix in the three batches of trial production express surface markers of mesenchymal stem cells, secrete a variety of growth factors, reduce the immune response, promote the formation of angiogenesis, and inhibit apoptosis of cardiomyocytes in vitro. Both the fresh and 24 h preserved UC−MSC sheets met the established quality standards.

## 2. Materials and Methods

### 2.1. Ethics Statements

Umbilical cord sample collection was approved by the OASIS International Hospital local ethics committee (No: LLPJ2018[001]). Written informed consent was obtained from the puerperas. Before parturition, puerperas should test negative for human immunodeficiency virus (HIV), human hepatitis B virus (HBV), human hepatitis C virus (HCV), *Treponema pallidum* (TP), human T-cell leukemia virus (HTLV), cytomegalovirus (CMV), and Epstein–Barr virus (EBV). Umbilical cord samples were preserved in sterile saline at 4 °C after collection and transferred to the laboratory within 24 h.

### 2.2. Cell Isolation and Culture

The human umbilical cord was washed with phosphate buffer saline (PBS) to remove blood. Then, blood vessels and tunica externa were removed, and Wharton’s jelly was separated. After being cut into small pieces, Wharton’s jelly was seeded into 100 mm Petri dishes coated with fetal bovine serum (FBS). Then, the Petri dishes were cultured at 37 °C, 5% CO_2_, and 95% humidity with umbilical cord mesenchymal stem cell (UC−MSC) medium consisting of minimum essential medium-α (α-MEM, Corning (Corning, NY, USA)), supplemented with 10% FBS (Gibco (Carlsbad, CA, USA)), 1% L-glutamine (Corning), 1% nonessential amino acid (NEAA, Gibco (Carlsbad, CA, USA)), and 20 IU/mL basic fibroblast growth factor (bFGF) (Langtai (Foshan, China)). UC−MSCs that migrated from the explants were defined as P0 UC−MSCs.

When reaching 90% confluence, UC−MSCs were detached using TrypLE (Gibco (Carlsbad, CA, USA)) and passaged to new flasks at a cell density of 2 × 10^4^ cells/cm^2^. P1 UC−MSCs cryopreserved at a density of 2 × 10^6^ cells/mL in cell freezing medium (FBS with 10% dimethyl sulfoxide (DMSO) (Sigma (St. Louis, MO, USA))) were defined as MCBs. UC−MSCs from the master cell bank (MCB) were recovered and passaged to P4 as mentioned above. Then, UC−MSCs at P4 were cryopreserved and defined as working cell banks (WCBs).

### 2.3. Cell Sheet Production and Preservation

UC−MSCs from the WCB were recovered and cultured to 100% confluence with UC−MSC medium. After being digested to single-cell suspension with TrypLE, UC−MSCs were washed three times with PBS to remove exogenous FBS and basic fibroblast growth factor (bFGF). Then, the UC−MSCs were suspended in a cell sheet-forming medium, which consisted of α-MEM supplemented with 0.1% human serum albumin (HSA) (Shandong Taibang, Taian, China). A total of 6 × 10^7^ cells were seeded into 100 mm temperature-responsive culture dishes (ThermoFisher (Waltham, MA, USA)) and cultured at 37 °C with 5% CO_2_ and 95% humidity overnight. Then, the UC−MSC sheet was detached from the temperature-responsive culture dishes at room temperature (20–25 °C).

After washing with saline solution, 5–10 mL of the corresponding UC−MSC sheet preservation solution was added to the UC−MSC sheet for preservation at 4 °C. The preservation solutions were: (1) normal saline solution with 1% HSA (Solution 1); (2) a commercial preservation solution, which is a kind of category III medical device (Solution 2); (3) Hypothermosol (Biolife Solutions (Bothell, WA, USA)) (Solution 3).

### 2.4. Cell Amount, Cell Viability, and Cell Apoptosis Assays

Cell amount and viability were evaluated by Acridine Orange and Propidium Iodide (AOPI) staining and automatic fluorescent cell counter (Countstar S2 (Shanghai, China)) detection, according to the manufacturer’s instructions. Cell apoptosis was tested with the Annexin V-FITC Apoptosis Detection Kit (eBioscience (Carlsbad, CA, USA)), according to the manufacturer’s instructions.

Samples were prepared as follows. For MCB and WCB cells, UC−MSCs were recovered and suspended in UC−MSC medium for detection. For UC−MSC sheets, the UC−MSC sheet was digested by TrypLE into single-cell suspension for detection.

### 2.5. Cell Growth Curve

UC−MSCs in MCB and WCB were recovered and seeded into 96-well plates at a concentration of 6000 cells/well in UC−MSC medium. Cell growth was evaluated each day by Cell Counting Kit-8 (CCK-8) assay (TransGen (Beijing, China)), according to the manufacturer’s instructions.

### 2.6. Cell Cycle Analysis

UC−MSCs in MCB and WCB were recovered and seeded in T75 flasks at a cell density of 2 × 10^4^ cells/cm^2^. When reaching 60–70% confluence, UC−MSCs were harvested, and the cell cycle was tested with the Cell Cycle Detection Kit (KeyGEN Biotech (Nanjing, China)), according to the manufacturer’s instructions.

### 2.7. Colony-Forming Unit (CFU) Assay

UC−MSCs in MCB and WCB were recovered and seeded in 96-well plates at a concentration of 1 cell/well. After culturing for 14 days, wells containing 50 or more cells were counted with a microscope (CKX41, OLYMPUS, Tokyo, Japan). The colony formation proportion is = the ratio of the number of wells containing 50 or more cells in/the number of total seeding wells.

### 2.8. Cell Surface Marker Detection

UC−MSCs in MCB and WCB were recovered and seeded in T75 flasks at a cell density of 2 × 10^4^ cells/cm^2^. When they reached 90–100% confluence, UC−MSCs were harvested for cell surface marker detection. For UC−MSC sheets, the sheet was digested using TrypLE into single-cell suspension for detection. UC−MSCs were aliquoted into 1 × 10^6^ cells/tube in the staining buffer, consisting of phosphate buffer saline (PBS, Corning (Corning, NY, USA)), supplemented with 1% FBS (Gibco). Then, anti-CD73-FITC (BD (Becton, NJ, USA)), anti-CD90-FITC (BD), anti-CD105-APC (BioLegend (San Diego, CA, USA)), anti-CD11b-FITC (BD), anti-CD19-FITC (BioLegend), anti-CD34-PE (BioLegend), anti-CD45-FITC (BD), anti-HLA-DR-FITC (BD), anti-IgG-FITC (BD), anti-IgG-PE (BD), and anti-IgG-APC (BD) were added to the tubes separately. After staining for 30 min at room temperature in the dark, the cells were washed twice with PBS and resuspended in the staining buffer for flow cytometry analysis.

### 2.9. Differentiation Assays

UC−MSCs in MCB and WCB were recovered and resuspended in UC−MSC medium for differentiation. For UC−MSC sheets, the cell sheet was digested by TrypLE into single-cell suspension for differentiation.

For adipogenic differentiation, cells were seeded in 24-well plates at a density of 8 × 10^4^ cells/well and cultured at 37 °C, 5% CO_2_, and 95% humidity. When the cells reached 100% confluence, the culture medium was changed to adipogenic differentiation medium (BI (Kibbutz Beit-Haemek, Israel)) to induce differentiation. After culturing for 14–21 days with medium change every three days, the cells were fixed and stained with an MSC Adipo-Staining Kit (VivaCell, Shanghai, China).

For osteogenic differentiation, cells were seeded in 24-well plates at a density of 4 × 10^4^ cells/well and cultured at 37 °C, 5% CO_2_, and 95% humidity. When the cells reached 70% confluence, the culture medium was changed to osteogenic differentiation medium (BI) to induce differentiation. After culturing for 14–21 days with medium change every three days, the cells were fixed and stained with an MSC Osteo-Staining Kit (VivaCell).

For chondrogenic differentiation, cells were seeded in 15 mL tubes at a density of 4 × 10^5^ cells/tube in chondrogenic differentiation medium (BI) as pellets and cultured at 37 °C, 5% CO_2_, and 95% humidity for 14–21 days with medium change every three days. The cells were fixed and stained with an MSC Chondro-Staining Kit (VivaCell).

### 2.10. Growth Factor Detection Assays

Cell banks: UC−MSCs in MCB and WCB were recovered and seeded in T75 flasks at a cell density of 2 × 10^4^ cells/cm^2^. Twenty-four hours before cell passage, the culture medium was replaced with 10 mL fresh UC−MSC medium. When passaging cells, the culture medium was collected and centrifuged at 300× *g* for 5 min to remove dead cells for growth factor detection. The number of viable cells was monitored using AOPI in the automatic fluorescent cell counter to calculate the amount of factor secretion per 1 × 10^6^ cells in 24 h.

Cell sheet-forming medium: Cell sheet-forming medium was collected and centrifuged at 300× *g* for 5 min to remove dead cells for growth factor detection.

Cell sheet reattachment medium: The freshly produced and 24 h preserved cell sheets were attached to 100 mm Petri dishes in UC−MSC medium and cultured for 24 h. The medium was collected and centrifuged at 300× *g* for 5 min to remove dead cells for growth factor detection. 

Hepatocyte growth factor (HGF) (Invitrogen (Carlsbad, CA, USA)), vascular endothelial growth factor (VEGF) (NOVUS (Centennial, CO, USA)), interleukin-8 (IL-8) (R&D (Minneapolis, MN, USA)), and interleukin-6 (IL-6) (NOVUS) were quantified using commercial enzyme-linked immunosorbent assay (ELISA) kits, following the manufacturer’s instructions.

### 2.11. High-Risk Substance Residue Detection Assays

The final cell washing solution before cell sheet fabrication was used for bFGF residue detection by following the manufacturer’s instructions for the Human bFGF ELISA Kit (Life Technologies (Carlsbad, CA, USA)).

Cell sheets were digested with 3 mL TrypLE, and the digestion was quenched with 7 mL PBS. The single-cell suspension was centrifuged at 300× *g* for 5 min, and the supernatant was harvested for human serum albumin (HSA) residue detection by following the manufacturer’s instructions for the Human Albumin ELISA Kit (BETHYL (Hamburg, Germany)).

Cell sheets were digested with 1 mL TrypLE supplemented with 50 μL 20% HSA, and the digestion was quenched with 1 mL cell sheet-forming medium. The single-cell suspension was centrifuged at 300× *g* for 5 min, and the supernatant was harvested for bovine serum albumin (BSA) and gentamicin residue detection by following the manufacturer’s instructions for the Bovine Albumin ELISA Kit (BETHYL) and GENTAMINCIN ELISA Kit (REAGEN (San Diego, CA USA)), respectively.

Cell sheets were harvested and lysed with 5 mL lysis buffer, which consisted of normal saline supplemented with protease inhibitor (Roche (Basel, Switzerland)), by a repeated liquid nitrogen freeze–thaw method. The lysate was centrifuged at 12,000× *g* for 20 min at 4 °C, and the supernatant was harvested for TrypLE and fibrinogen residue detection by following the manufacturer’s instructions for the TrypLE ELISA Kit (JunYan (Guangzhou, China)) and Human Fibrinogen ELISA Kit (Novus (Centennial, CO, USA)), respectively.

The residual amount of bFGF is presented in pg/mL. The residual amounts of BSA, HSA, gentamicin, TrypLE, and fibrinogen are presented in ng per cell sheet.

### 2.12. Immunoregulatory Test Assay

UC−MSCs in MCB and WCB were recovered and seeded in 6-well plates at a density of 5 × 10^5^ cells/well in UC−MSC medium. Cell sheets were digested as described in the “cell amount, cell viability and cell apoptosis assays” and seeded in 6-well plates at a density of 5 × 10^5^ cells/well in UC−MSC medium. After overnight culture, the cells were treated with 10 μg/mL mitomycin to inhibit division. Then, human peripheral blood mononuclear cells (PBMCs) were recovered and seeded into wells with or without UC−MSCs in PBMC medium, which consisted of Roswell Park Memorial Institute 1640 (RPMI 1640) (Gibco (Carlsbad, CA, USA)) supplemented with 10% FBS, 1% L-glutamine, and 1% NEAA, at a density of 1 × 10^6^ cells/well.

For Th1 lymphocyte detection, PBMCs were co-cultured with UC−MSCs overnight. Cell Stimulation Cocktail (plus protein transport inhibitors) (500X) (eBioscience (Carlsbad, CA, USA)) was added to the wells for Th1 lymphocyte activation, according to the manufacturer’s descriptions. PBMCs co-cultured with UC−MSCs without activation were used as the negative control. PBMCs cultured alone with activation were used as the positive control. Then, the proportions of Th1 lymphocytes were detected by anti-CD3-APC (BD), anti-CD8-FITC (BD), and anti-IFNγ-PE (BD) staining. A minimum of 10,000 events were acquired on a BD FACS Canto II flow cytometer, and the proportions of CD3^+^CD8^−^IFNγ^+^ Th1 lymphocytes were analyzed using the FlowJo VX software (BD (Becton, NJ, USA)).

For lymphocyte proliferation detection, 10 μg/mL phytohemagglutinin M (PHA-M) (Sigma (St. Louis, MO, USA)) was added to activate PBMCs co-cultured with UC−MSCs. PBMCs co-cultured with UC−MSCs without activation were used as the negative control. PBMCs cultured alone with PHA-M activation were used as the positive control. After 3 days of co-culturing, 10 μM BrdU was added to each well overnight. Then, the PBMCs were harvested and stained with the BrdU Staining Kit (eBioscience (Carlsbad, CA, USA)), according to the manufacturer’s instructions. A minimum of 10,000 events were acquired on a BD FACS Canto II flow cytometer, and the proportions of BrdU-positive proliferating lymphocytes were analyzed using the FlowJo software.

For TNFα detection, the culture medium in the lymphocyte proliferation detection before BrdU staining was harvested for tumor necrosis factor α (TNFα) detection by the Human TNFα ELISA Kit (Invitrogen (Carlsbad, CA, USA)), according to the manufacturer’s descriptions.

### 2.13. Angiogenesis Assay

Human umbilical vein epithelial cells were seeded in 48-well plates pre-coated with 100 μL Matrigel (BD) at a density of 1000 cells/well. Conditioned cell sheet-forming medium was added to the wells and cultured for 12 h. Fresh cell sheet-forming medium was used as the negative control. Images were taken using an inverted microscope and analyzed using the angiogenesis analyzer.

### 2.14. Cardiomyocyte Apoptosis Inhibition Assay

H9C2 rat cardiomyocytes were seeded in 6-well plates at a density of 15,000 cells/cm^2^. When the cells reached 80% confluence, 300 μM CoCl_2_ was added to the culture medium for 24 h to induce H9C2 apoptosis. Conditioned cell sheet-forming medium was added to the wells and cultures for 24 h. Fresh cell sheet-forming medium was used as the negative control. Cell apoptosis was tested with the Annexin V-FITC Apoptosis Detection Kit (eBioscience), according to the manufacturer’s instructions.

### 2.15. Immunofluorescence Staining

UC−MSC sheets were fixed with 4% paraformaldehyde and embedded in the optimal cutting temperature compound (Sakura Finetek (Tokyo, Japan)). After being cut into 10 μm sections using a cryostat (Leica), the cell sheets were blocked for 1 h at room temperature with blocking buffer, which consisted of PBS supplemented with 2% BSA. They were then labeled with primary antibodies against fibronectin (Abcam) or integrin β1 (Abcam) at 4 °C overnight, followed by incubation with FITC-conjugated donkey anti-rabbit antibody (Jackson ImmunoResearch (West Grove, PA, USA)) for visualization at room temperature for 2 h. Nuclei were counterstained with Hoechst (Life Technologies (Carlsbad, CA, USA)) at room temperature for 10 min. Then, the sections were assessed by confocal laser-scanning microscopy.

### 2.16. STR Authentication

UC−MSCs in MCB and WCB and cell sheets were prepared as described in the “cell amount, cell viability and cell apoptosis assay”. One million cells were collected and used for short tandem repeat (STR) authentication by the fluorescence STR method. The detected gene loci included D5S818, D13S317, D7S820, D16S539, VWA, TH01, TPOX, CSF1PO, D3S1358, Penta E, D2S441, D2S1338, Penta D, D10S1248, D19S433, D21S11, D18S51, D6S1043, D8S1179, D12S391, and FGA.

### 2.17. Telomerase Activity Detection

UC−MSCs in MCB and WCB and cell sheets were prepared as described in the “Cell amount, cell viability and cell apoptosis assay” section. One million cells were collected and used for telomerase activity detection with the fluorescence real-time quantitative PCR detection kit for telomerase activity (human) (KeyGEN BioTECH (Shanghai, China)), according to the manufacturer’s instructions.

### 2.18. Sterility Testing

The culture method was used for sterility testing. Briefly, cell sheet was digested with 3 mL TrypLE (Gibco (Carlsbad, CA, USA)) at 37 °C for 3 min. After quenching the digestion with 7 mL PBS, 3 mL of the cell suspension was used for sterility testing. For MCB and WCB cells, UC−MSCs were thawed, and 3 mL of the cell suspension was used for sterility testing. After membrane filtration and culture for 14 days, the results were evaluated.

### 2.19. Mycoplasma Detection

Cell culture supernatant before cryopreservation and cell sheet-forming supernatant before cell sheet detachment were tested by the culture method and indicated cell culture method.

For the quantitative polymerase chain reaction (Q-PCR) method, cells in MCB, WCB, and cell sheets were prepared as described in the “Cell amount, cell viability and cell apoptosis assay” section. One million cells were collected and used for mycoplasma detection with the Mycoplasma DNA Extraction and Purification Kit (magnetic bead method) and Mycoplasma DNA Detection Kit (PCR-Fluorescent Probe Method), both from Houzhou Shenke (Huzhou, China), according to the manufacturer’s instructions.

### 2.20. Endotoxin Test

Cell sheets were digested as described in “Sterility testing”, and the digestion supernatant was used for endotoxin test by the Gel Clot LAL Assay.

### 2.21. Exogenous Virus Detection

Exogenous virus was tested both in vitro and in vivo.

For in vitro different indicator cell inoculation and culture methods, monkey-derived Vero cells, human-derived MRC-5 cells, and human MSC cells were used as indicator cells.

For in vivo methods, mouse intraperitoneal and intracerebral vaccination, suckling mouse intraperitoneal and intracerebral vaccination, 5–6 days of chicken embryo yolk sac vaccination, and 9–11 days of chicken embryo allantois vaccination were conducted.

### 2.22. Bovine Virus Detection

The cell culture and fluorescent antibody detection methods were used for bovine virus detection.

### 2.23. Human Virus Detection

Either cell culture supernatant or one million cells was used for HIV, HBV, HCV, CMV, EBV, TP, Human cell virus B19 (HB19), and Human spore virus type 6 (HHV-6) detection using the following kits according to the manufacturer’s instructions: Human Immunodeficiency Virus Type I (HIV-1) Nucleic Acid Quantitative Determination Kit (PCR-Fluorescence Probe Method), Hepatitis B virus nucleic acid determination kit (PCR fluorescent probe method), Hepatitis C virus nucleic acid determination kit (PCR fluorescent probe method), Human cytomegalovirus nucleic acid quantitative detection kit (PCR fluorescence method), EB virus nucleic acid amplification (PCR) fluorescence quantitative detection kit, Treponema pallidum nucleic acid detection kit (fluorescence PCR method), Human cell virus B19 nucleic acid detection kit (PCR-fluorescent probe method), and Human spore virus type 6 nucleic acid detection kit (PCR-fluorescent probe method). The HIV, HBV, HCV, CMV, EBV, HB19, and HHV-6 kits were from Zhongshan Da’an (Guangzhou, China), and the TP kit was from Suzhou Tianlong (Suzhou, China).

### 2.24. Retroviral Detection

Product-enhanced reverse transcriptase assay (PERT) method was used for retroviral detection.

## 3. Results

### 3.1. Developing the UC−MSC Sheet Production Process Conforming to Drug Standards

In the cell sheet formation process, the culture medium and Petri dish coating matrix are two key factors. For clinical safety concerns, the culture medium and coating matrix should be Xeno-free and exclude research-grade human extracts or exogenous growth factors.

After large-scale screening, we developed a safe cell sheet-forming medium with simple composition, containing only basic cell culturing medium (α-MEM) and a certain concentration of human serum albumin (HSA) (parenteral drug). For the coating matrix, fibrinogen, a component of the human fibrin sealant kit (drug), was chosen. Furthermore, the concentration of HSA in the cell sheet-forming medium and fibrinogen in the coating buffer was further optimized for stable and highly efficient cell sheet production. The results showed that α-MEM supplemented with 0.1% HSA as the cell sheet-forming medium and 10 μg/mL fibrinogen as the coating buffer could support umbilical cord mesenchymal stem cell (UC−MSC) sheet formation (data not shown). 

**Figure 1:**
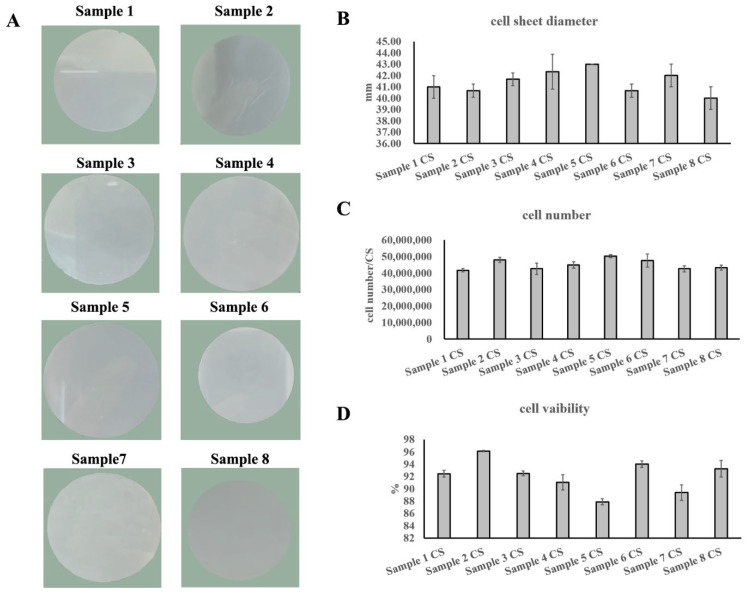
The umbilical cord mesenchymal stem cell (UC−MSC) sheet production process conformation. (**A**) Morphology of the UC−MSC sheets. (**B**) Statistics of diameters of the UC−MSC sheets. (**C**) Statistical analysis of total cell amount per UC−MSC sheet. (**D**) Statistical analysis of cell viability of the UC−MSC sheets.

Then, eight UC−MSC lines from eight individuals were used to fabricate UC−MSC sheets following the optimized production process, all of which were able to successfully fabricate UC−MSC sheets with the appearance of a white round sheet structure, smooth surface, and neat edges (Figure 1A). The diameter of the UC−MSC sheets was between 39 mm and 43 mm (Figure 1B). The Acridine Orange and Propidium Iodide (AOPI) staining results showed that the cell amount of one UC−MSC sheet was between 40 and 50 million (Figure 1C). The cell viability of all eight batches of UC−MSC sheets was more than 85% (Figure 1D). All of these showed that the UC−MSC sheet production process developed here was stable with a broad spectrum, which could be used for large-scale UC−MSC sheet production.

### 3.2. Developing the UC−MSC Sheet Preservation Process Conforming to Drug Standards

After production, the UC−MSC sheet products need to be transported to the clinical site that will administer the cells, thus the UC−MSC sheet preservation method and solution need to be developed. Through theoretical analysis, three kinds of preservation solutions were chosen, which included: (1) normal saline solution with 1% HSA (Solution 1); (2) a commercial preservation solution, which is a kind of category III medical device (Solution 2); (3) Hypothermosol (Biolife Solutions) (Solution 3).

**Figure 2:**
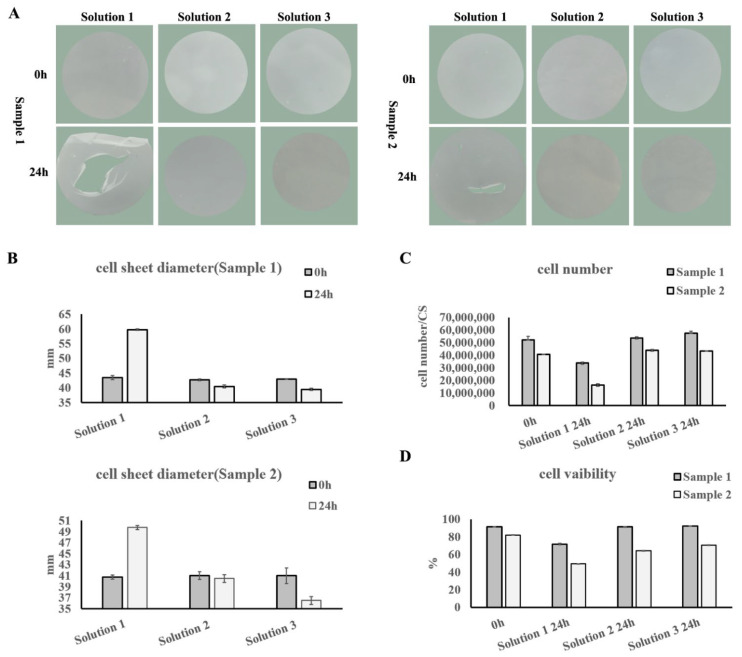
The UC−MSC sheet preservation solution selection. (**A**) Morphology of the UC−MSC sheet. (**B**) Statistics of cell sheet diameters. (**C**) Statistical analysis of total cell amount per UC−MSC sheet. (**D**) Statistical analysis of cell viability of the UC−MSC sheets.

UC−MSC sheets were kept in the corresponding preservation solution at 4 °C. The appearance, diameter, total cell amount, and cell viability of the UC−MSC sheet were determined at 0 h and 24 h (Figure 2). After 24 h, no structural damage occurred and there was no obvious change in diameter in Solution 2 and Solution 3 (Figure 2A,B). While the structure was damaged, the diameter significantly increased after being stored in Solution 1 for 24 h (Figure 2A,B). As for total cell amount and cell viability, no differences were detected between Solution 2 and Solution 3, but both were higher than that of Solution 1 (Figure 2C,D).

As a result, the preservation effect of Solution 2 and Solution 3 was comparable, and better than Solution 1. As a research-grade preservation solution, the ingredients of Solution 3 were disclosed, which have great safety and compliance risks. Solution 2 was a Class III medical device with clear ingredients and contents and low safety and compliance risks. Therefore, Solution 2 was chosen as the UC−MSC sheet preservation solution. 

### 3.3. Establishment of Quality Standards of the UC−MSC Sheet

As a cell therapy product, quality standards and release quality standards should be defined. Based on our research foundations and the “ICH Q5A: Viral Safety Evaluation of Biotechnology Products Derived from Cell Lines of Human or Animal Origin”, “ICH Q6B: Test Procedures and Acceptance Criteria for Biotechnological/Biological Products”, “Guiding Principles for Quality Control of Stem Cell Preparations and Preclinical Research”, “Guidelines for Cell Therapy Products and Evaluation Technology”, and the regulations for MSC of the International Society for Cell Therapy (ISCT) [18], quality standards of the UC−MSC sheet involving product characteristics, safety, and functionality were formulated. By comprehensively considering the necessity, representativeness, and effectiveness of the testing items, the release quality standards were selected from the total quality standards. Please refer to Table 1 for details.

### 3.4. Large-Scale Production of UC−MSC Sheets

For UC−MSC sheet production, all processes, including the acquisition of umbilical cords and the production of intermediate and final products, should be managed in accordance with GMP regulations. Established standard operation procedures (SOPs) and standard record procedures (SRPs) should be strictly implemented in all processes. Intermediate products should meet the established quality standards before they can enter the next production stage.

For the acquisition of umbilical cords, donors with informed consent should have no family history and no serious infectious diseases, including human immunodeficiency virus (HIV), human hepatitis B virus (HBV), human hepatitis C virus (HCV), *Treponema pallidum* (TP), human T-cell leukemia virus (HTLV), cytomegalovirus (CMV), Epstein–Barr virus (EBV), and SARS-CoV-2 infection. The umbilical cord should be tested according to our established quality control items and standards (Table 2) before primary cell isolation.

To ensure the safety, effectiveness, and quality of the UC−MSC sheet final products, the two-tiered cell banks including the master cell bank (MCB) and working cell bank (WCB) were established as intermediate products. The qualified MCBs were used to establish WCBs. The qualified WCBs were the final cell sources for UC−MSC sheet production. For the quality standards of MCB and WCB, please refer to Table 3.

**Figure 3:**
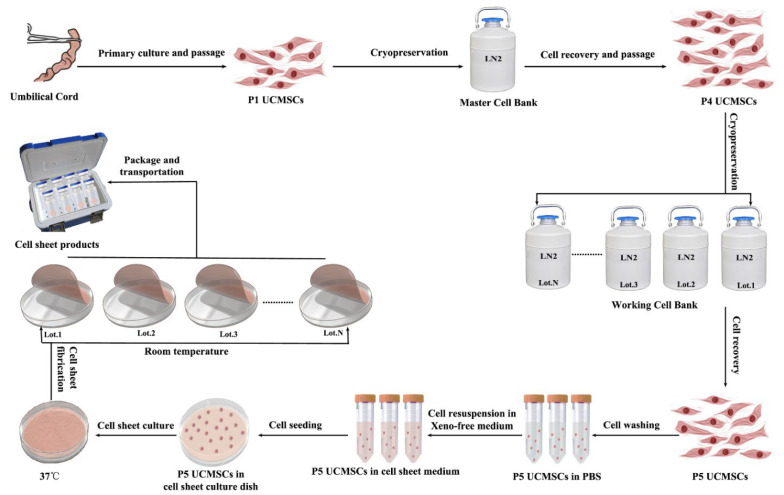
Schematic diagram of the large-scale production of UC−MSC sheets.

According to the established processes, one umbilical cord could be used to establish one batch of MCB; one batch of MCB could be used to establish several batches of WCBs; one batch of WCB could be used to produce several batches of cell sheet products (Figure 3). Therefore, human UC−MSC sheet products can be mass-produced to ensure safety, effectiveness, and quality.

According to the established large-scale UC−MSC sheet production process, three batches of MCBs, with more than 300 million cells in each MCB, were successfully established from three umbilical cords. Three batches of WCBs, with more than 2400 million cells in each WCB, were successfully established from three independent batches of MCBs. Then, three batches of UC−MSC sheet final products were successfully produced (trial) from three independent batches of WCBs. The amount of UC−MSC sheet produced in each batch was 35 sheets, which could be used for more than 10 patients in future clinical trials.

### 3.5. Identification of MCB and WCB

Both the established MCBs and WCBs were tested according to items listed in Table 3 and shown to meet the quality standards. The cell morphology of all UC−MSCs in the MCBs and WCBs that adhered to plastic culture dishes was a spindle shape (Supplementary Figure S1A). The cell growth curves showed that all UC−MSCs had high proliferation activity (Supplementary Figure S1B). Cell density, cell viability, and cell apoptosis were all tested and shown to meet the quality standards (Supplementary Figure S1C–E). The UC−MSCs were positive for the cell surface markers CD73, CD90, and CD105 (positive cell proportion ≥ 95%) and negative for the cell surface markers CD11b, CD19, CD34, CD45, and HLA-DR (positive cell proportion ≤ 2%) (Table 4). Cell cycle assays showed that UC−MSCs had a typical diploid cell cycle (Supplementary Figure S1F). The Colony-Forming Unit (CFU) results showed that UC−MSCs could form single-cell colonies (Supplementary Figure S1G). In addition, UC−MSCs could differentiate into osteoblasts, adipocytes, and chondroblasts (Supplementary Figure S1H) under appropriate induction conditions.

For functionality, UC−MSCs in MCBs and WCBs secreted Hepatocyte growth factor (HGF) interleukin-6 (IL-6) and interleukin-8 (IL-8) (Supplementary Figure S2). HGF and IL-6 can promote angiogenesis, and IL-6 can reduce immunogenicity [19,20]. UC−MSCs in all the MCBs and WCBs inhibited lymphocyte proliferation, downregulated the secretion of tumor necrosis factor α (TNFα), and inhibited lymphocyte differentiation into the Th1 subtype (Supplementary Figure S3). All these results showed that UC−MSCs have immunomodulatory effects.

For the safety assays, three batches of MCBs and WCBs were tested for bacteria, fungi, and mycoplasma, and the results were all negative. No telomerase activity, which is high in fast proliferating cells such as pluripotent stem cells and cancer cells, was detected in the three batches of MCBs and WCBs. The short tandem repeat (STR) authentication results showed that the three batches of MCBs and WCBs all had a single peak at the 21 tested sites, and the peaks of the corresponding MCBs and WCBs were the same, indicating that no cell cross-contamination occurred in the cell bank construction. As the MCBs were not directly used for cell sheet product fabrication, some safety tests were only conducted in WCBs. No pathogenic microorganisms, including bovine virus, which may be introduced by FBS in the UC−MSC medium, or retrovirus and human HIV, HBV, HCV, CMV, EBV, TP, Human cell virus B19 (HB19), and Human spore virus type 6 (HHV-6), which may be introduced by the raw material umbilical cord and throughout the entire production process, were detected in the three batches of WCBs. No colony formation in soft agar was detected in the three batches of WCBs, indicating that the cells had no tumorigenicity. Additionally, the three batches of WCBs all had normal karyotypes, further proving that the cells had no risk of tumorigenicity. The methods of the safety test items are listed in Table 3, and the results are listed in Table 5.

### 3.6. Identification of UC−MSC Sheets

For the three trial batches of UC−MSC sheets, some were tested immediately after production, and some were tested after 24 h of storage in Solution 2. The test items and quality standards are listed in Table 1.

**Figure 4:**
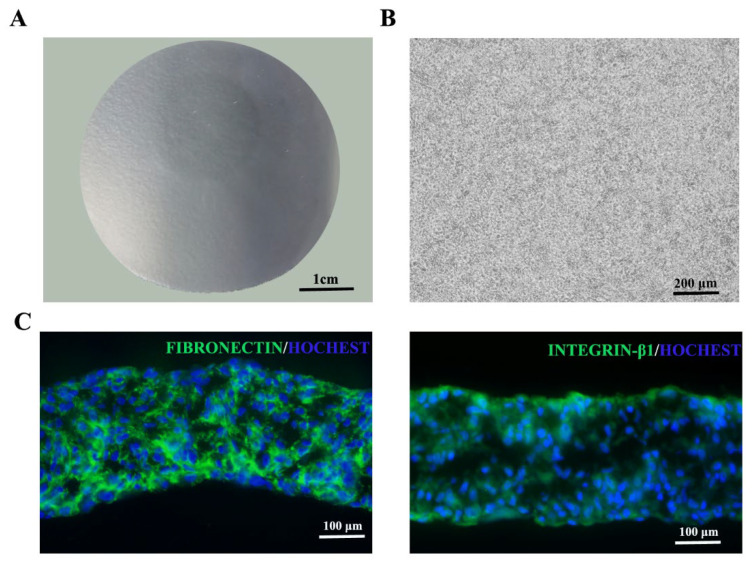
Structure of the UC−MSC sheet. (**A**) Morphology of the UC−MSC sheet. (**B**) Enlarged morphology of the UC−MSC sheet. (**C**) Fibronectin and Integrin β1 staining of the UC−MSC sheet.

For physical and chemical properties, the three batches of UC−MSC sheets tested were all white round sheet structures with smooth surfaces and neat edges (Figure 4A). Morphological observation under the microscope showed tight cell connections in the UC−MSC sheets (Figure 4B). Immunofluorescence results showed that the UC−MSC sheet reserved intact extracellular matrix fibronectin and integrin β1 (Figure 4C).

**Figure 5:**
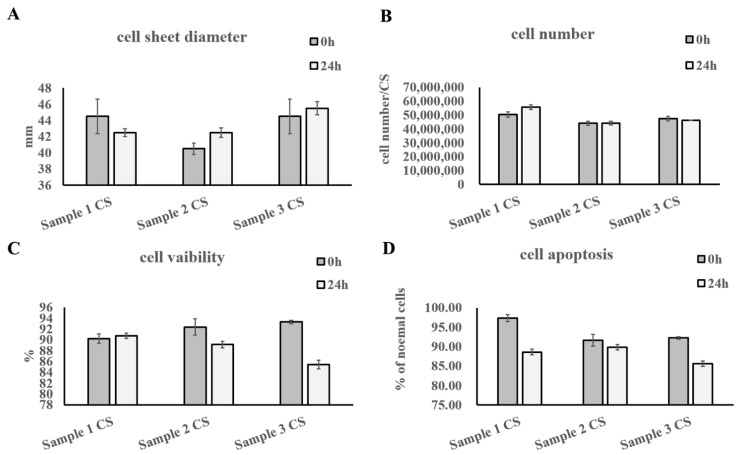
Characteristic tests of UC−MSC sheets. (**A**) Statistics of cell sheet diameters. (**B**) Statistical analysis of cell amount per sheet. (**C**) Statistics of cell viability. (**D**) Statistical analysis of cell apoptosis.

The diameters of the freshly produced and 24 h preserved UC−MSC sheets were between 35 and 50 mm (Figure 5A). The cell amount per UC−MSC sheet was between 30 and 60 million (Figure 5B). The AOPI staining results showed that the cell viability of freshly produced and 24 h preserved UC−MSC sheets was more than 70% (Figure 5C). The cell apoptosis analysis showed results similar to those of AOPI staining (Figure 5D).

**Figure 6:**
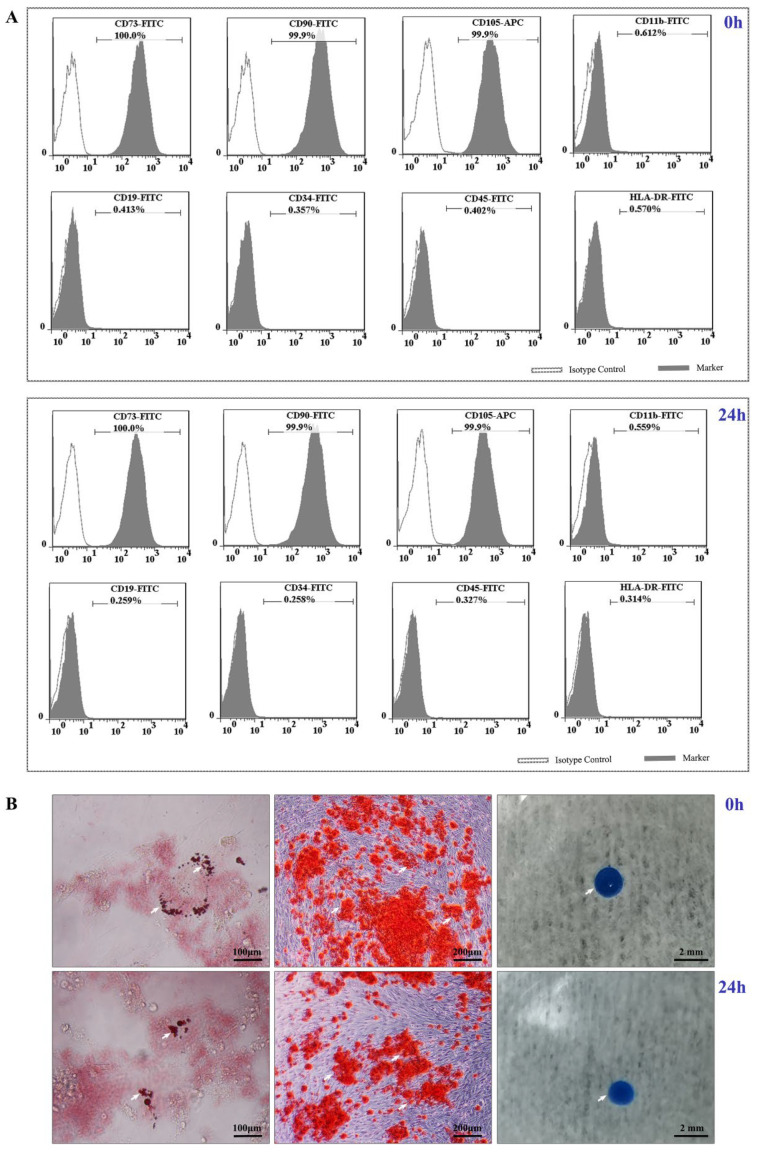
Cell characteristic tests of UC−MSC sheets. (**A**) Cell surface marker expression of freshly and 24 h preserved cell sheets. (**B**) Representative results of induced adipogenic (white arrows point to lipid droplets stained with oil red), osteogenic (white arrows point to calcium nodules stained with alizarin red), and chondrogenic (white arrows point to cartilage stained with alcian blue) differentiation of freshly and 24 h preserved cell sheets.

UC−MSCs in both freshly produced and 24 h stored cell sheets were positive (positive cell proportion ≥ 95%) for the cell surface markers CD73, CD90, and CD105 and negative (positive cell proportion ≤ 2%) for the cell surface markers CD11b, CD19, CD34, CD45, and HLA-DR (Figure 6A and Table 6) and could be induced to differentiate into osteoblasts, adipocytes, and chondroblasts (Figure 6B).

**Figure 7:**
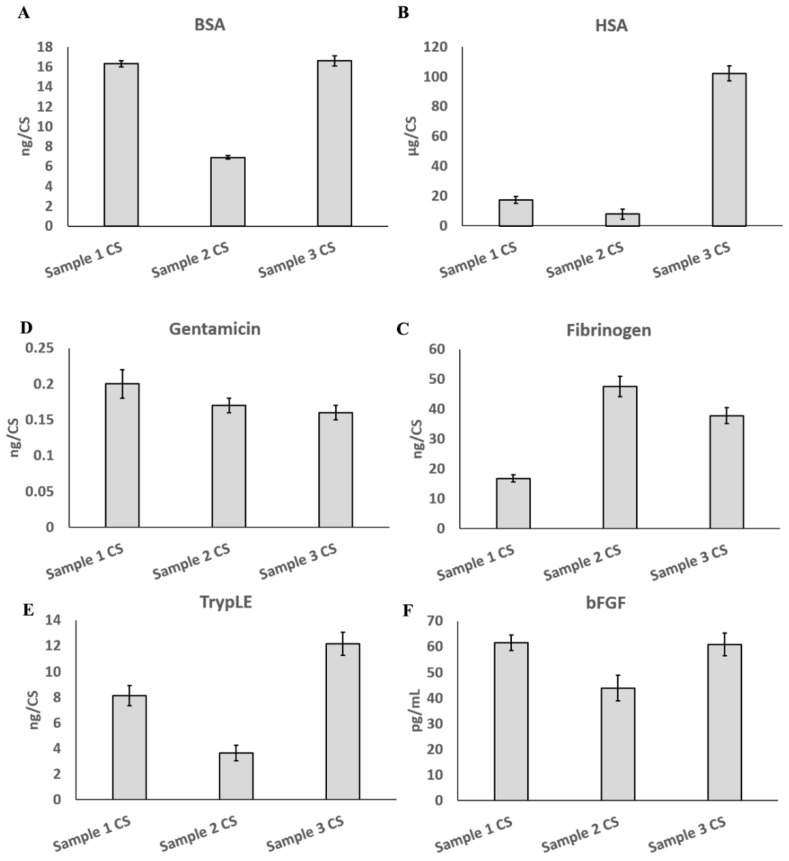
Detection of residues of high-risk substances. (**A**) Bovine serum albumin (BSA). (**B**) Human serum albumin (HSA). (**C**) Gentamicin. (**D**) Fibrinogen. (**E**) TrypLE. (**F**) Basic fibroblast growth factor (bFGF).

Residues of high-risk substances, including bovine serum albumin (BSA), human serum albumin (HSA), gentamicin, fibrinogen, TrypLE, and basic fibroblast growth factor (bFGF), used in the production process were detected and controlled. The quality standards are listed in Table 1. The test results showed that all three batches of the UC−MSC sheets satisfied the quality standards (Figure 7).

**Figure 8:**
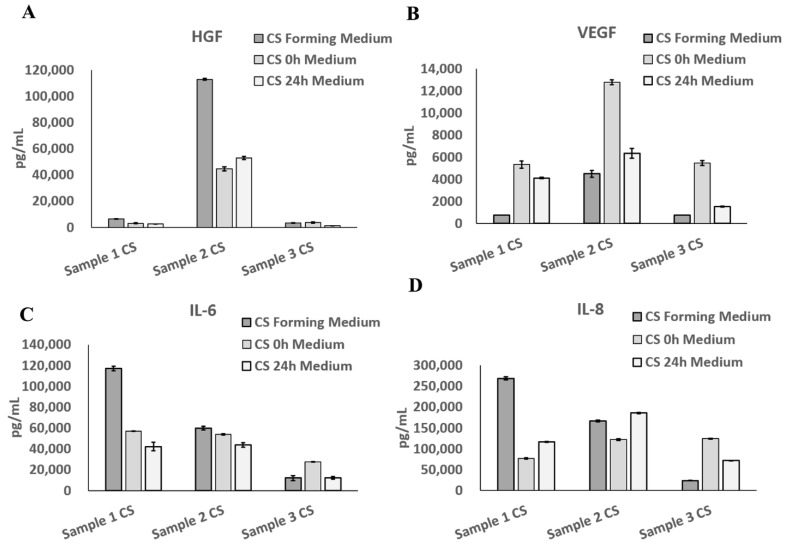
Growth factor detection of UC−MSC sheet. (**A**) Hepatocyte growth factor (HGF) (**B**) Vascular endothelial growth factor (VEGF) (**C**) Interleukin-6 (IL-6) (**D**) Interleukin-8 (IL-8).

UC−MSC sheets took effect by secreting growth factors, which could promote microvessel formation, promote tissue repair, and perform immune regulation. HGF, vascular endothelial growth factor (VEGF), and IL-8 can promote microvessel formation; IL-6 can reduce immunogenicity, and IL-8 and IL-6 can promote immunoregulation [19,20]. The UC−MSC sheet could be reattached to the culture dish in UC−MSC medium in vitro to imitate its state in the body. All three batches of the UC−MSC sheet could secrete HGF, VEGF, IL-6, and IL-8 in the cell sheet-forming process and the freshly produced and 24 h preserved UC−MSC sheet reattachment process (Figure 8).

**Figure 9:**
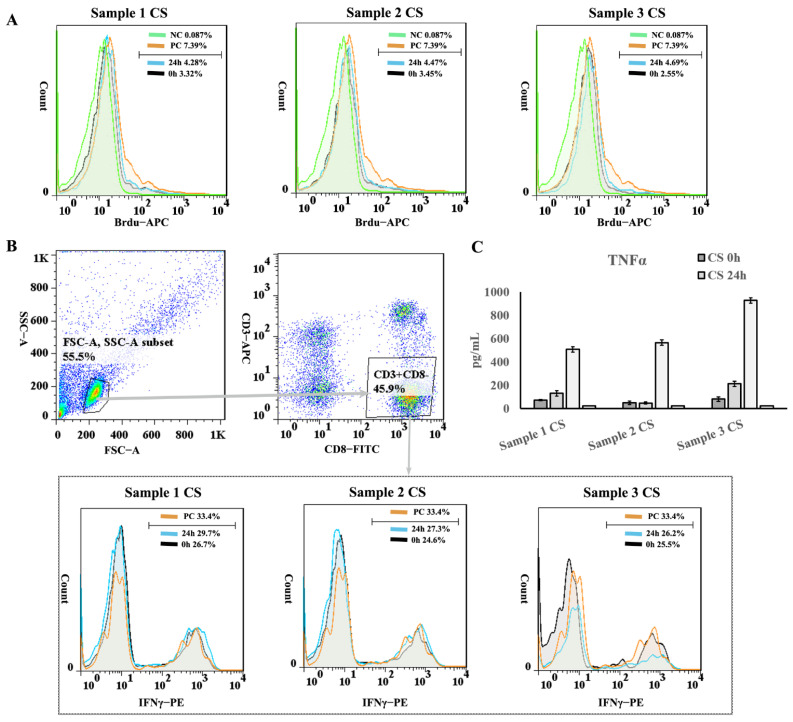
Immunomodulatory effect test of the UC−MSC sheet. (**A**) Detection of lymphocyte proliferation inhibition ability of UC−MSC sheet. (**B**) Detection of lymphocyte tumor necrosis factor α (TNFα) secretion inhibition ability of UC−MSC sheet. (**C**) Detection of Th1 lymphocyte inhibition ability of UC−MSC sheet.

When co-cultured with activated human peripheral blood mononuclear cells (PBMCs), UC−MSC sheets inhibited lymphocyte proliferation, Th1 subtype differentiation, and inflammatory factor TNFα secretion, demonstrating their immunomodulatory effect in vitro (Figure 9).

**Figure 10:**
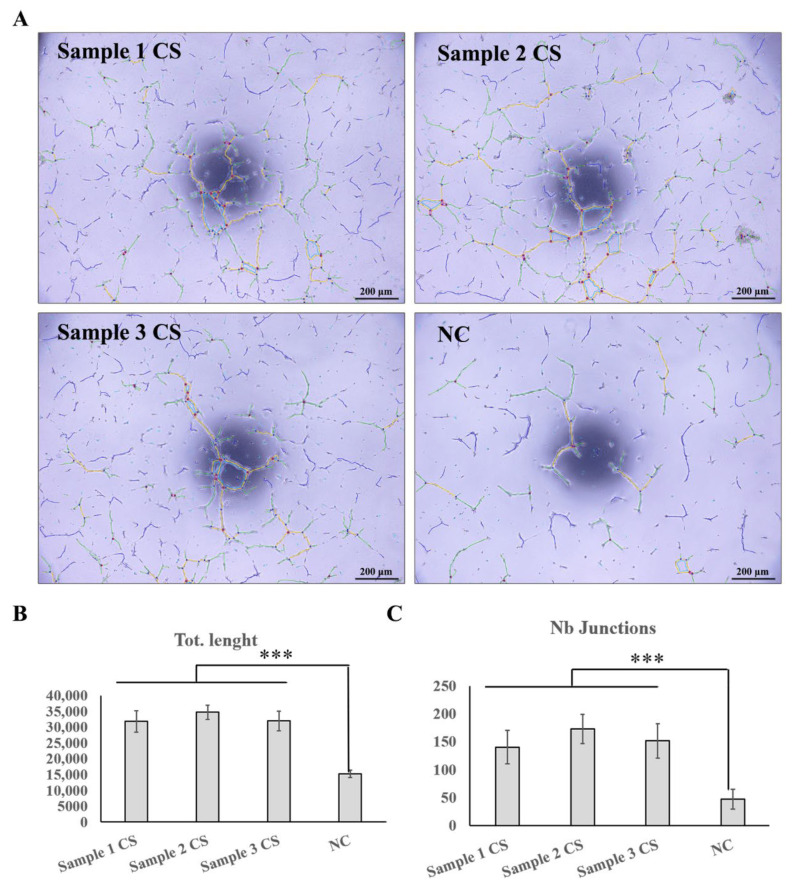
Proangiogenic function test of UC−MSC sheet. (**A**) ImageJ Angiogenesis Analyzer was used to quantify endothelial tube formation. Images were taken using an inverted microscope and analyzed using an angiogenesis analyzer. Yellow lines represent tubes that connect together to different junctions; green lines represent branches; dark blue represents isolated tubes; red circles represent the master junction points. (**B**) Statistical results of Nb junctions. (**C**) Statistical results of total length. *** represent *p* < 0.01.

Conditioned cell sheet-forming medium increased the total length of microtubules and the number of microtubule junctions in the angiogenesis formation model in vitro, demonstrating its angiogenesis formation promoting ability (Figure 10).

**Figure 11:**
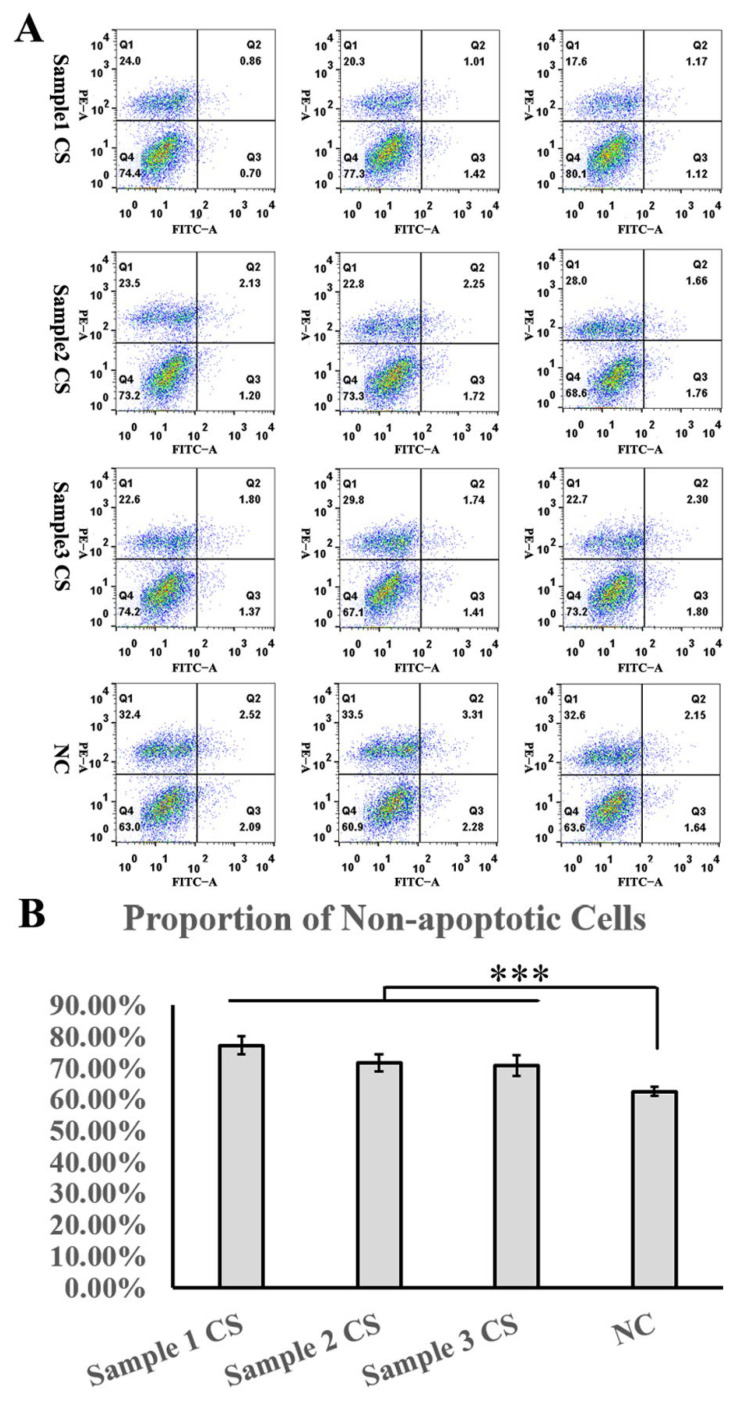
Anti-apoptotic function test of the cell sheet. (**A**) Apoptosis results of H9C2 cells cultured in cell sheet-forming conditioned medium or negative control medium after induction of apoptosis by cobalt chloride. (**B**) Statistical results of apoptosis results of H9C2 cells cultured in cell sheet-forming conditioned medium or negative control medium after apoptosis was induced by cobalt chloride. *** represent *p* < 0.01.

Additionally, the conditioned cell sheet-forming medium inhibited cobalt chloride-induced apoptosis of rat cardiomyocytes in vitro, demonstrating its cell repair ability in vitro (Figure 11).

For the safety assays, all three batches of freshly produced and 24 h stored UC−MSC sheets were negative for bacteria, fungi, and mycoplasma. The STR authentication results showed that the three batches of UC−MSC sheets all had a single peak at the 21 tested sites, and the peaks were the same as the corresponding MCBs and WCBs, indicating that no cell cross-contamination occurred in the cell sheet production process. Gel Clot LAL Assays were used for the endotoxin test. The results showed that all three batches of freshly produced and 24 h stored UC−MSC sheets were less than or equal to 6.6 EU per cell sheet, which satisfied the quality standard. Please refer to Table 7 for details.

## 4. Discussion

To meet the needs of clinical trials for product safety, quality consistency, and large quantity, we developed the production and preservation technology of umbilical cord mesenchymal stem cell (UC−MSC) sheets and established a large-scale production and multi-level quality control system conforming to Good Manufacturing Practice (GMP) regulations, which is in preparation for clinical trials of UC−MSC sheet products in the development stage of new drugs and which could lay the foundation for industrial large-scale production after the new drug is launched. 

For cell sheet production, the culture medium and Petri dish coating matrix are two key factors. Due to the high density and multilayer cells in the cell sheet, the residual culture medium and coating matrix cannot be eliminated thoroughly by the cell sheet postharvest washing procedures. The safety and efficacy of UC−MSC sheets may be significantly influenced by the chosen media and Petri dish coating matrix.

According to the GMP guidelines and cell therapy drug standards, the safe cell sheet-forming medium with simple composition, containing only a basic cell culturing medium (minimum essential medium-α (α-MEM)) and a certain concentration of human serum albumin (HSA) (parenteral drug), and the safe coating matrix, fibrinogen, a component of the human fibrin sealant kit (drug), were developed for cell sheet production in this study. Therefore, the cell sheet products are safe and GMP compliant.

The preparation of cell sheets requires a large number of cells, in the range of 20–100 million UC−MSCs/sheet. To meet the huge demand for expanded cells, expansion processes need to be in place. In fact, exogenous growth factor basic fibroblast growth factor (bFGF), a spray drug for external use, and fetal bovine serum (FBS), a research-grade animal extract, were still used in the cell culture process. Before cell sheet fabrication, FBS and bFGF were removed by constant washing and controlled in the final cell sheet products. Ideally, the culture medium in the whole process of cell sheet manufacture, including cell culture, should be chemically defined and not contain animal extracts, research-grade human extracts, or research-grade exogenous growth factors. Although several serum-free media have been investigated for human mesenchymal stem cell (MSC) expansion [21,22,23,24], ingredients of these mediums could not be risk-evaluated and controlled in final products due to their confidentiality, which does not meet the GMP guidelines and drug standards. In addition, these media contain a variety of research-grade exogenous growth factors or human/animal extracts, which would also cause safety risks for clinical applications. This is also our next challenge. We will further optimize the cell expansion medium to realize risk-free clinical safety in the manufacturing process.

To meet the needs of clinical trials for large quantity and quality consistency of the product, we standardized the production process of UC−MSC sheets on a large scale, including donor screening, cell bank establishment and quality control, and cell sheet production and quality control in this study. However, these are linear scale-ups based on strict production process control and strict quality control, including the UC−MSC culture process and UC−MSC sheet production process. The current production process cannot meet the needs of large-scale production after the product is put on the market. Industrial UC−MSC expansion and UC−MSC sheet production systems need to be developed. Now, research on large-scale cell expansion by automation equipment, which could monitor changes in various key parameters, such as pH and temperature, during cell culture is on the way and has been utilized in some cell types [25,26,27,28,29]. Moreover, the Quantum^®^ equipment has been used for MSC expansion [30]. Based on the GMP-compliant UC−MSC expansion and quality control systems in this study and the research foundations of automation cell expansion equipment, industrial large-scale expansion of UC−MSCs that could be used for UC−MSC sheet production will soon be realized. As for cell sheet fabrication, Ayako and colleagues researched an equipment that could allow 10 cell sheets to be simultaneously cultured in parallel [31]. Based on the equipment and our UC−MSC sheet fabrication standard operation procedures (SOPs) and quality control systems, we will develop an automated cell sheet fabrication equipment for larger-scale UC−MSC sheet fabrication to meet the needs of new drug launches in the future. 

Following production, the cell sheet products need to be transported to the clinical site that will administer the products. Although the cell sheet preservation technology, which was compliant with the GMP practice, developed in this study confirmed the safety of the cell sheets in the process of transportation and preservation before clinical use, limitations still existed. The validity period of the UC−MSC sheets was only 24 h, which limits the time for cell sheet transplantation for clinical application. Therefore, further study is needed to develop long-term preservation or freezing technology for cell sheets, including cryopreservation in liquid nitrogen technology. Several teams have successfully cryopreserved epithelial cell sheets [32,33]. When thawed, the epithelial cell sheets maintained a tissue repair function [33]. With the development of tissue cryopreservation technology, cryopreserving mesenchymal stem cell sheets would soon be realized. 

UC−MSC sheets produced and preserved according to the process described in this study express surface markers of mesenchymal stem cells, secrete a variety of growth factors, reduce the immune response, promote the formation of microvasculature, and inhibit apoptosis of cardiomyocytes in vitro. For the safety of UC−MSC sheets, our other study showed that there was no tumor formation, and the cell sheets only existed in the transplanted tissues or organs in Severe Combined Immunodeficient (SCID) mice [34]. For the function of UC−MSC sheets, the research results showed UC−MSC sheets could obviously improve left ventricular heart function, and ejection fraction (EF) improved from 30% to 50% in mouse infarction models [10] and from 40% to 67% in porcine infarction models [34]. These all indicated that the UC−MSC sheets in this study could be used as a cell therapy drug in future clinical trials after being administrated by the National Medical Products Administration.

## 5. Conclusions

The quality and safety of UC−MSC sheets must be controlled to ensure their final use in patients. This study has developed a preparation and preservation technology for clinical-grade serum-free UC−MSC sheets that is safe and effective and provides application prospects for cell therapy.

## Figures and Tables

**Table 1:** Quality standards of UC−MSC sheet.

Categories	Items	Methods	Quality Standards
Physical and chemical properties	* Cell sheet appearance	Visual observation	White round sheet structure with smooth surface and neat edges
	* Cell sheet diameter	Ruler method	35–50 mm
	* Cell amount per cell sheet	Acridine Orange and Propidium Iodide (AOPI) staining and automatic fluorescent cell counter detection	3–6 × 10^7^ cells/cell sheet
	* Cell viability	AOPI staining and automatic fluorescent cell counter detection	≥70%
	* Cell apoptosis detection	Annexin V and Propidium Iodide (PI) staining and flow cytometry detection	Normal cells ≥ 70%
Biological characteristics	* Cell surface marker CD73 detection	Fluorescent antibody staining and flow cytometry detection	Positive cell proportion ≥ 95%
	* Cell surface marker CD90 detection	Fluorescent antibody staining and flow cytometry detection	Positive cell proportion ≥ 95%
	* Cell surface marker CD105 detection	Fluorescent antibody staining and flow cytometry detection	Positive cell proportion ≥ 95%
	* Cell surface marker CD11b detection	Fluorescent antibody staining and flow cytometry detection	Positive cell proportion ≤ 2%
	* Cell surface marker CD19 detection	Fluorescent antibody staining and flow cytometry detection	Positive cell proportion ≤ 2%
	* Cell surface marker CD34 detection	Fluorescent antibody staining and flow cytometry detection	Positive cell proportion ≤ 2%
	* Cell surface marker CD45 detection	Fluorescent antibody staining and flow cytometry detection	Positive cell proportion ≤ 2%
	* Cell surface marker HLA-DR detection	Fluorescent antibody staining and flow cytometry detection	Positive cell proportion ≤ 2%
	Induced osteoblast differentiation	Induce differentiation and alizarin red staining	Positive for alizarin red staining
	Induced adipocyte differentiation	Induce differentiation and Oil Red O staining	Positive for Oil Red O staining
	Induced chondroblast differentiation	Induce differentiation and alcian blue staining	Positive for alcian blue staining
Biological functions	Lymphocyte proliferation inhibition test	Co-culture with peripheral blood mononuclear cells (PBMCs), BrdU staining, and flow cytometry detection	Inhibit lymphocyte proliferation
	Th1 lymphocyte inhibition test	Co-culture with PBMCs, fluorescent antibody staining, and flow cytometry detection	Inhibit Th1 lymphocyte differentiation
	Inflammatory factor- tumor necrosis factor α (TNFα) secretion inhibition test	Co-culture with PBMCs and enzyme-linked immunosorbent assay (ELISA) detection	Inhibit TNFα secretion
	* Secretory factor vascular endothelial growth factor (VEGF) detection	ELISA	Report result
	* Secretory factor hepatocyte growth factor (HGF) detection	ELISA	Report result
	Secretory factor interleukin-6 (IL-6) detection	ELISA	Report result
	Secretory factor interleukin-8 (IL-8) detection	ELISA	Report result
Safety	* STR authentication	Fluorescence short tandem repeat (STR) method	Single peak at detection sites
	* Gentamicin residue detection	ELISA	≤0.5 ng/cell sheet
	* Bovine serum albumin (BSA) residue detection	ELISA	≤16.6 ng/cell sheet
	* Human serum albumin (HSA) residue detection	ELISA	Report result
	* Basic fibroblast growth factor (bFGF) residue detection	ELISA	≤100 pg/mL
	Fibrinogen residue detection	ELISA	Report result
	* TrypLE residue detection	ELISA	≤16.6 ng/cell sheet
	* Sterility testing	Membrane filtration method	Negative
	* Mycoplasma detection	Culture method	Negative
	* Mycoplasma detection	Indicated cell culture method	Negative
	* Mycoplasma detection	quantitative polymerase chain reaction (Q-PCR)	Negative
	* Endotoxin test	Gel Clot LAL Assay	≤6.6 EU/cell sheet

* Release quality standards.

**Table 2:** Quality standards of umbilical cord.

Categories	Items	Methods	Quality Standards
Character	Appearance	Naked eye observation	Complete packaging without leakage
	Temperature	Thermometry	2–8 °C
	Umbilical cord collection information	Naked eye observation	Complete
	Within the warranty period	Naked eye observation	Less than 24 h from umbilical cord collection
Safety	human immunodeficiency virus (HIV)	Colloidal gold method	Negative
	human hepatitis B virus (HBV)	Colloidal gold method	Negative
	human hepatitis C virus (HCV)	Colloidal gold method	Negative
	*Treponema pallidum* (TP)	Colloidal gold method	Negative
	human T-cell leukemia virus (HTLV)	ELISA	Negative
	Epstein–Barr virus (EBV)	ELISA	Negative
	cytomegalovirus (CMV)	Colloidal gold method	Negative
	SARS-CoV-2	Q-PCR	Negative

**Table 3:** Quality standards of MCB and WCB.

Categories	Items	Methods	Quality Standards
Physical and chemical properties	Cell morphology	Microscopic observation	Adherent and spindle-shaped cells
	Cell amount	AOPI staining and automatic fluorescent cell counter detection	1.6–2.4 × 10^6^/mL
	Cell viability	AOPI staining and automatic fluorescent cell counter detection	≥80%
	Cell apoptosis detection	Annexin V and PI staining and flow cytometry detection	Normal cells ≥ 80%
Biological characteristics	Cell growth curve	CCK-8 assay	Report result
	Cell cycle analysis	PI staining and flow cytometry detection	Report result
	Colony-Forming Unit (CFU)	Single-cell culture in 96-well plates	Report result
	Cell surface marker CD73 detection	Fluorescent antibody staining and flow cytometry detection	Positive cell proportion ≥ 95%
	Cell surface marker CD90 detection	Fluorescent antibody staining and flow cytometry detection	Positive cell proportion ≥ 95%
	Cell surface marker CD105 detection	Fluorescent antibody staining and flow cytometry detection	Positive cell proportion ≥ 95%
	Cell surface marker CD11b detection	Fluorescent antibody staining and flow cytometry detection	Positive cell proportion ≤ 2%
	Cell surface marker CD19 detection	Fluorescent antibody staining and flow cytometry detection	Positive cell proportion ≤ 2%
	Cell surface marker CD34 detection	Fluorescent antibody staining and flow cytometry detection	Positive cell proportion ≤ 2%
	Cell surface marker CD45 detection	Fluorescent antibody staining and flow cytometry detection	Positive cell proportion ≤ 2%
	Cell surface marker HLA-DR detection	Fluorescent antibody staining and flow cytometry detection	Positive cell proportion ≤ 2%
	Induced osteoblast differentiation	Induce differentiation and alizarin red staining	Positive for alizarin red staining
	Induced adipocyte differentiation	Induce differentiation and Oil Red O staining	Positive for Oil Red O staining
	Induced chondroblast differentiation	Induce differentiation and alcian blue staining	Positive for alcian blue staining
Biological functions	Lymphocyte proliferation inhibition test	Co-culture with PBMCs, BrdU staining, and flow cytometry detection	Inhibit lymphocyte proliferation
	Th1 lymphocyte inhibition test	Co-culture with PBMCs, fluorescent antibody staining, and flow cytometry detection	Inhibit Th1 lymphocyte differentiation
	Inflammatory factor-TNFα secretion inhibition test	Co-culture with PBMCs and ELISA detection	Inhibit TNFα secretion
	Secretory factor HGF detection	ELISA	Report result
	Secretory factor IL-6 detection	ELISA	Report result
	Secretory factor IL-8 detection	ELISA	Report result
Safety	STR authentication	Fluorescence STR method	Single peak at detection sites
	Telomerase activity detection	Q-PCR	Negative
	Sterility testing	Membrane filtration method	Negative
	Mycoplasma detection	Culture method	Negative
	Mycoplasma detection	Indicated cell culture method	Negative
	Mycoplasma detection	Q-PCR	Negative
	* HIV-1 detection	Q-PCR	Negative
	* HBV detection	Q-PCR	Negative
	* HCV detection	Q-PCR	Negative
	* HCMV detection	Q-PCR	Negative
	* EBV detection	Q-PCR	Negative
	* TP detection	Q-PCR	Negative
	* Human cell virus B19 (HB19) detection	Q-PCR	Negative
	* Human spore virus type 6 (HHV-6) detection	Q-PCR	Negative
	* Retroviral detection	Product-enhanced reverse transcriptase assay (PERT) method	Negative
	* Bovine virus detection	Cytopathic observation method	Negative
	* Bovine virus detection	Hemoabsorption test	Negative
	* Bovine virus detection	Fluorescent antibody test	Negative
	* Exogenous virus detection	Vero cell culture test	Normal cell morphology
	* Exogenous virus detection	MRC-5 cell culture test	Normal cell morphology
	* Exogenous virus detection	Human MSC culture test	Normal cell morphology
	* Exogenous virus detection	Vero cell erythrocyte adsorption and blood coagulation test	Negative
	* Exogenous virus detection	MRC-5 cell erythrocyte adsorption and blood coagulation test	Negative
	* Exogenous virus detection	MSC erythrocyte adsorption and blood coagulation test	Negative
	* Exogenous virus detection	Mouse intraperitoneal and intracerebral vaccination	Negative
	* Exogenous virus detection	Suckling mouse intraperitoneal and intracerebral vaccination Chinese Pharmacopoeia	Negative
	* Exogenous virus detection	5–6 day chicken embryo yolk sac vaccination	Negative
	* Exogenous virus detection	9–11 day chicken embryo allantoic vaccination	Negative
	* Exogenous virus detection	Erythrocyte adsorption test of 9–11 days chicken embryo allantoic fluid	Negative
	* Karyotype analysis	G-band method	46XX or 46XY
	* Tumorigenicity test	Soft Agar Clone Formation Experiment	Negative

* Only tested in WCB.

**Table 4:** Cell surface marker test of cell banks.

Markers	Positive Cell Proportion					
	Sample 1		Sample 2		Sample 3	
	MCB	WCB	MCB	WCB	MCB	WCB
CD73	99.7%	99.7%	99.8%	99.9%	99.3%	99.9%
CD90	99.9%	99.8%	99.8%	99.9%	99.8%	99.8%
CD105	99.8%	99.9%	99.8%	99.5%	99.9%	99.9%
CD11b	0.191%	0.020%	0.050%	0.080%	0.060%	0.210%
CD19	0.142%	0.070%	0.120%	0.220%	0.190%	0.240%
CD34	0.122%	0.020%	0.040%	0.060%	0.140%	0.140%
CD45	0.145%	0.050%	0.050%	0.050%	0.070%	0.070%
HLA-DR	0.997%	0.080%	0.050%	0.290%	0.260%	0.280%

**Table 5:** Safety test of MCB and WCB.

Items	Results					
	Sample 1		Sample 2		Sample 3	
	MCB	WCB	MCB	WCB	MCB	WCB
Telomerase activity detection	Negative	Negative	Negative	Negative	Negative	Negative
Sterility testing	Negative	Negative	Negative	Negative	Negative	Negative
Mycoplasma detection	Negative	Negative	Negative	Negative	Negative	Negative
HIV detection	N/A	Negative	N/A	Negative	N/A	Negative
HBV detection	N/A	Negative	N/A	Negative	N/A	Negative
HCV detection	N/A	Negative	N/A	Negative	N/A	Negative
CMV detection	N/A	Negative	N/A	Negative	N/A	Negative
EBV detection	N/A	Negative	N/A	Negative	N/A	Negative
TP detection	N/A	Negative	N/A	Negative	N/A	Negative
HB19 detection	N/A	Negative	N/A	Negative	N/A	Negative
HHV-6 detection	N/A	Negative	N/A	Negative	N/A	Negative
Retrovirus detection	N/A	Negative	N/A	Negative	N/A	Negative
Bovine virus detection	N/A	Negative	N/A	Negative	N/A	Negative
Exogenous virus detection	N/A	Negative	N/A	Negative	N/A	Negative
STR authentication	Single peak at detection sites	Single peak at detection sites	Single peak at detection sites	Single peak at detection sites	Single peak at detection sites	Single peak at detection sites
Karyotype analysis	N/A	46 XX	N/A	46 XY	N/A	46 XY
Tumorigenicity test	N/A	Negative	N/A	Negative	N/A	Negative

**Table 6:** Cell surface marker test of the UC−MSC sheets.

Markers	Positive Cell Proportion					
	Sample 1		Sample 2		Sample 3	
	0 h	24 h	0 h	24 h	0 h	24 h
CD73	100.0%	100.0%	99.8%	99.9%	99.9%	99.9%
CD90	99.9%	99.9%	99.9%	99.9%	99.9%	99.8%
CD105	99.9%	99.9%	99.9%	99.5%	99.6%	99.9%
CD11b	0.612%	0.559%	0.485%	0.916%	0.027%	0.031%
CD19	0.413%	0.259%	0.273%	0.516%	0.074%	0.031%
CD34	0.357%	0.258%	0.231%	0.131%	0.170%	0.015%
CD45	0.402%	0.327%	0.172%	0.538%	0.009%	0.016%
HLA-DR	0.570%	0.314%	0.252%	0.324%	0.064%	0.008%

**Table 7:** Safety test of the UC−MSC sheet.

Items	Methods	Results		
		Sample 1	Sample 2	Sample 3
Sterility test	Membrane filtration method	Negative	Negative	Negative
Mycoplasma test	Q-PCR method	Negative	Negative	Negative
Mycoplasma test	Culture method	Negative	Negative	Negative
STR authentication	Fluorescence STR method	Single peak at detection sites	Single peak at detection sites	Single peak at detection sites
Endotoxin test	Gel Clot LAL Assay	≤6.6EU/CS	≤6.6EU/CS	≤6.6EU/CS

## Data Availability

The datasets used and analyzed during the current study are available from the corresponding author upon reasonable request.

## Supplementary Materials

The following supporting information can be downloaded at: https://www.mdpi.com/article/10.3390/cells11172732/s1, Figure S1: Cell characteristic test of the MCBs and WCBs; Figure S2: Growth factor detection of the MCBs and WCBs; uppl3: Immune modulating function test of the MCBs and WCBs.
