# Supplementary material for: Manufacture and Quality Control of Human Umbilical Cord-Derived Mesenchymal Stem Cell Sheets for Clinical Use
Source: Cells. 2022 Sep 1;11(17):2732. doi: 10.3390/cells11172732 (PMC9454431; doi:10.3390/cells11172732)
Supplement: Supplementary file 1 [file cells-11-02732-s001.zip › cells-1836955-supplementary.pdf]

cells

MDPI

Supplementary Material

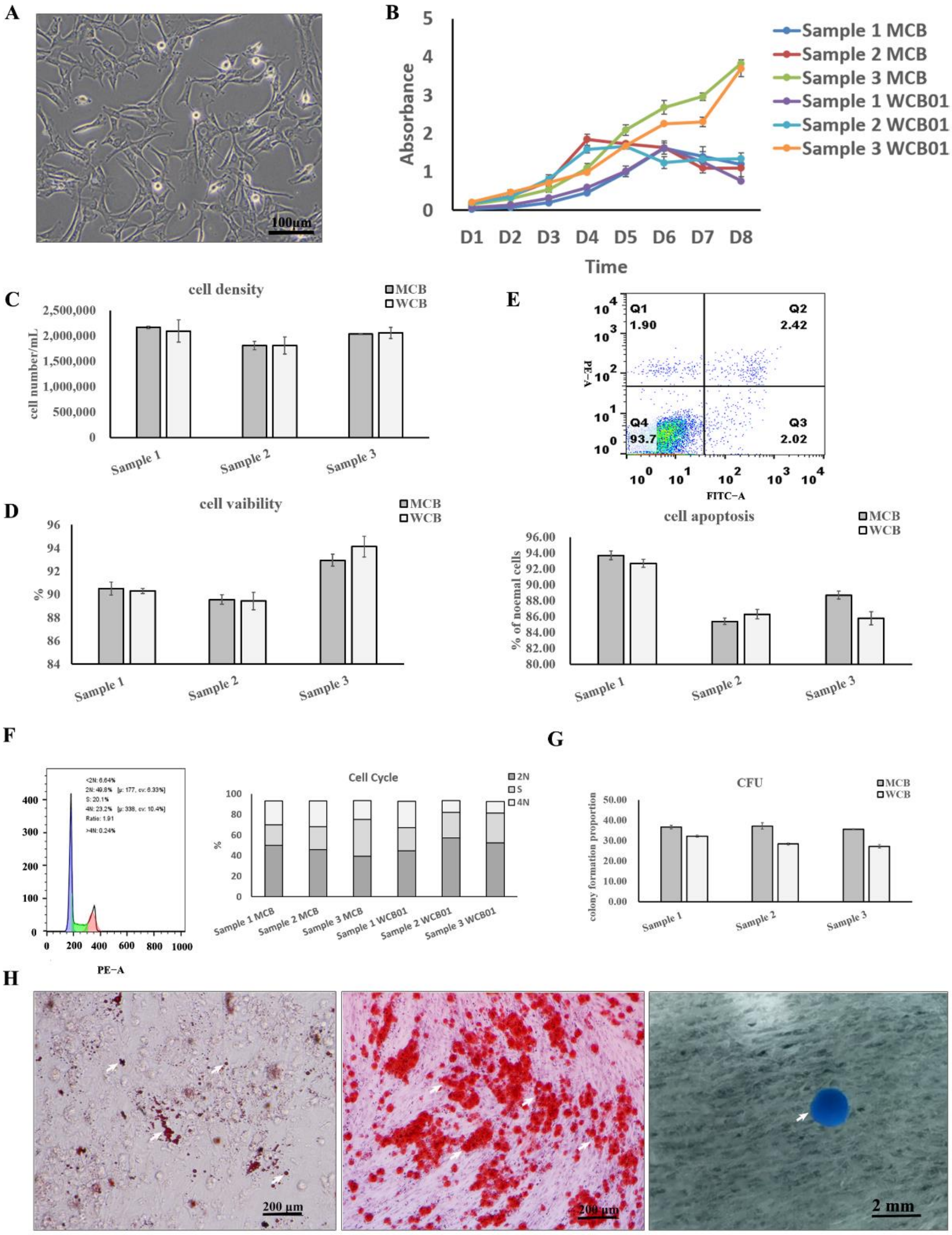

Figure S1. Cell characteristic test of the MCBs and WCBs. (**A**) Cell morphology of HUC-MSCs. (**B**) Cell growth curve of the MCBs and WCBs. (**C**) Cell density of the MCBs and WCBs. (**D**) Cell viability of the MCBs and WCBs. (**E**) Cell apoptosis of MCBs and WCBs. (**F**) Cell cycle of the MCBs and WCBs. (**G**) Colony formation proportions of the MCB and WCBs. (**H**) Representative results of induced adipogenic, osteogenic and chondrogenic differentiation of the MCBs and WCBs.

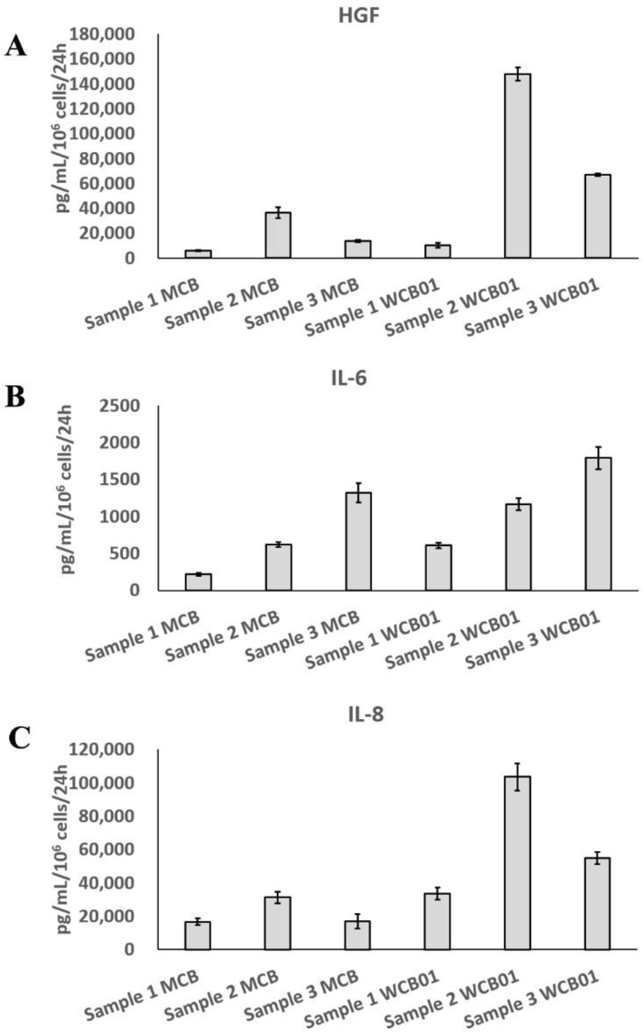

Figure S2. Growth factor detection of the MCBs and WCBs. (**A**) HGF. (**B**) IL-6. (**C**) IL-8.

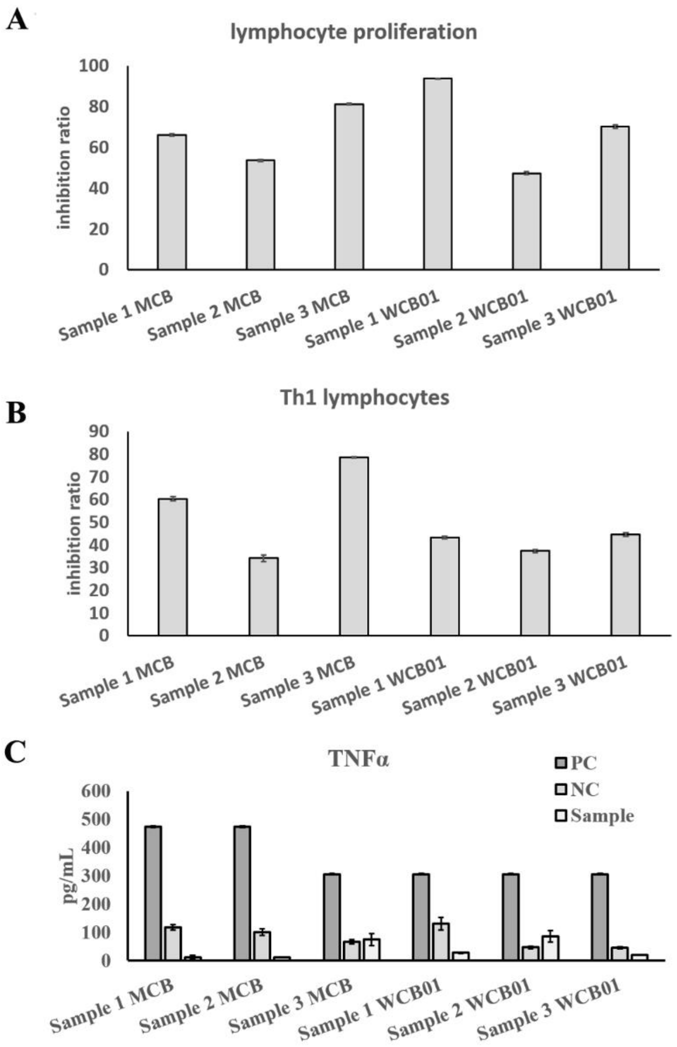

Figure S3. Immune modulating function test of the MCBs and WCBs. (**A**) Detection of lymphocyte proliferation inhibition ability of the MCBs and WCBs. (**B**) Detection of Th1 lymphocyte inhibition ability of the MCBs and WCBs. (**C**) Detection of lymphocyte TNFα secretion inhibition ability of the MCBs and WCBs.
